# Wired to Doubt: Why People Fear Vaccines and Climate Change and Mistrust Science

**DOI:** 10.3389/fmed.2021.809395

**Published:** 2022-01-28

**Authors:** Geoffrey P. Dobson

**Affiliations:** Heart and Trauma Research Laboratory, College of Medicine and Dentistry, James Cook University, Townsville, QLD, Australia

**Keywords:** science, truth, climate change, social media, education, coronavirus, vaccine hesitancy, denialists

## Abstract

We all want to be right in our thinking. Vaccine hesitancy and global warming denial share much in common: (1) both are threats to personal, community and global health, (2) action is contingent on co-operation and social policy, and (3) public support relies on trust in science. The irony is, however, as the science has become more convincing, public opinion has become more divided. A number of early polls showed that ~70% of people supported COVID-19 vaccine use and global warming, ~20% adopted a wait-and-see approach, and ~10% were staunch objectors. Although these percentages are approximate, what factors are responsible for the differences in engagement, doubt and distrust? How can we reduce the consensus gap? One approach is to return to grass roots and provide a brief history of the issues, understand the difference between fact and opinion, truth and falsehood, the problem of certainty, and how scientific consensus is reached. To doubt is a healthy response to new information, and it too has a scientific basis. Doubt and distrust reside in that region of the brain called the dorsolateral prefrontal cortex, which is responsible for suppressing unwanted representations. Bridging the consensus gap requires shifting human thinking patterns from doubt to belief, and belief to action. Education and improved public messaging are key, and social media providers require urgent oversight or regulation to remove false and harmful/dangerous content from our digital lives. Delays to vaccinate and failure to reduce greenhouse gases will dramatically change the way we live. The new norm may be more deadly COVID variants, strained healthcare systems, extreme weather patterns, diminished food supply, delays in goods and services, damage to world's economies and widespread global instability.

## Introduction

Everyone is entitled to his own opinion, but not to his own facts.

Daniel P. Moynihan (1983)

In early 2019, the World Health Organization (WHO) declared that vaccine hesitancy and climate change were major threats to global health (1). Imagine for a moment watching or listening to a debate or interview on vaccine hesitancy or climate change. Two leaders present opposing viewpoints. Time is short. One argues vaccines are safe and effective, and the other argues they are not safe, with specific examples. Similarly, in another debate a global warming advocate argues that human activity is responsible for increasing greenhouse emissions, and the opposing side argues the CO_2_ increases are part of a natural cycle. Both opponents claim the scientific evidence is undecided and conflicting, which fosters uncertainty and doubt. How does the public decide who is right and who is wrong? How do we separate fact from opinion? By definition, facts are considered true based on the preponderance of evidence, whereas opinions are not necessarily so (Box 1). After presenting a brief history of the science of vaccination and climate change, we will address the problem of certainty, examine the inner workings of science, discuss the science of doubt and distrust, and provide possible solutions on how to bridge the current gaps.

Box 1 **Definitions.**

**Social media**: refer to the different electronic-mediated technologies or platforms where users create and share content, and their profiles, with other people or groups as part of social networking and dissemination of knowledge. The largest social media networks are Facebook, Instagram, Twitter, YouTube, and TikTok, and have over 4 billion users (see text).

**Vaccine hesitancy**: is the delay in acceptance or refusal of vaccination despite the wide availability of vaccination services (2, 3).

**Climate change denial**: is a viewpoint that rejects the linkage between burning of fossil fuels, CO_2_ rise and global warming, which opposes the scientific consensus (4).

**Conspiracy theories**: are unsubstantiated explanations of events or circumstances that contradict current rational or scientific consensus, and are against the common good (5, 6). Theories claiming that vaccine or climate scientists purposely fake their data to receive research funding, or that COVID-19 and climate change are hoaxes perpetrated by government, are examples of conspiracy theories. They are easy to propagate and difficult to refute because they perpetuate doubt and mistrust.

**Greenhouse gases**: CO_2_, methane, nitrous oxide and chlorofluorocarbons are examples of greenhouse gases. These gases absorb radiation from the Earth's surface, reflect it back and warm its surface (7). A garden greenhouse works the same way: the sun's light energy is absorbed by plants and objects and converted to heat. Much of the trapped heat can't escape and warms the house. Heat is controlled by windows, vents or fans.

**Facts**: are propositions or statements that are considered true based on the preponderance of evidence established by unbiased, objective and verifiable (or falsifiable) methods. Facts may change as more evidence is obtained and knowledge advances. The term “alternative fact” is misleading and designed to create confusion and delegitimize what is true (8).

**Opinions**: are statements that may or may not be factual or supported by evidence. Opinions are generally personal beliefs or from like-minded friends or social media groups.

**Difference between fact and opinion**: depends upon the type and quality of verifiable (or falsifiable) evidence to support them (see above).

**Brain plasticity**: is a term that refers to the brain's ability to change and adapt as a result of experience, injury or disease, and involves building of new or existing circuitry.

**Fake news**: is made-up information that is patently false, which has become a major phenomenon in the context of Internet-based media (8). Although “fake news” spiked after the 2016 US Presidential election, it is not new. It dates back at least 400 years to Francis Bacons' *Novum Organum* (1620), where he discussed it as a deliberate obstruction of understanding, which was later termed “confirmational bias” (9) p179. Today, fake news or stories are created to deliberately misinform or deceive readers or manipulate users on a variety of topics (see Conspiracy Theories above) (8, 10).

**Science:** is a process of learning and understanding about the physical world through observation, measurement, experiment and prediction. The process is driven by curiosity and wonder, and its results are always under scrutiny. Science's provisional basis drives the process.

**Scientific explanation**: is an explanation about the physical world that involves facts, conceptual schemes and predictions that are testable (or falsifiable) (11).

**Truth:** is an elusive concept and comes in many forms; truths may be scientific, religious, mathematical, environmental, evolutionary, cultural, social, moral, ethical, legal, political, and so on. They are the building blocks of the facts and opinions we hold, and our worldview (see text). Different worldviews may generate different set of truths that correspond to different boundary assumptions, assertions, correspondence and coherence (11).

**Worldview**: A worldview is a collection of personal, family, religious and cultural attitudes, values, stories and expectations about the world we live in (11). It shapes how we view science, religion, public health policy and social media, as well as our willingness to believe in conspiracy theories, attitudes toward authority and accepting public consensus.

## Vaccine Hesitancy: It'S Nothing New

Resistance to these laws (small-pox vaccination) began immediately after passage of the 1853 law, with violent riots in Ipswich, Henley, Mitford, and several other towns. The founding of the Anti-Vaccination League in London in the same year provided a nucleus for opponents of vaccination.

Wolfe RM and LK Sharp (12)

It is an indisputable fact that vaccines are one of the greatest achievements of science and public health policy (2, 3). Every year, vaccinations save around 4–5 million people globally (13). Vaccine hesitancy dates back over 250 years when the smallpox (variola virus) epidemic swept through Europe, including France (1762–1763) (14). Despite a 30–40% chance of dying with no treatment, a number of Parisian doctors questioned the safety of inoculations, which was supported by the public (14, 15). Inoculations (or variolations) were used for centuries before the vaccine era and comprised tiny volumes of *live* infected matter to stimulate the body to protect against smallpox, and other infectious diseases (16). The inoculator used a lancet to transfer fresh matter taken from a ripe pustule of a person infected with smallpox (16).

In response to medical and public concerns, Paris's leading court issued an order halting the practice in 1763, and requested the *Faculty of Medicine* to opine on its safety and efficacy (14, 15). A debate ensued with one side arguing inoculations may cause death, and the other side claiming that they may save lives (14). This was a good debate. In the end, the received advice was to educate people to make informed decisions, not to mandate inoculations (14). In contrast, across the English channel, Reverend Edmund Massey in 1772 delivered a sermon and called these smallpox inoculations “diabolical operations”, “that was an attempt to oppose God's punishments upon man for his sins” (15).

The modern vaccine era began around 1800 with the development of smallpox vaccine by Edward Jenner (1749–1823) (16, 17). After years of research on cowpox, and hearing stories that dairymaids were protected from smallpox *after having suffered from cowpox*, Jenner injected mild dead virus prepared from a young dairymaid, Sarah Nelms, who had fresh cowpox lesions, into 8-year-old James Phipps (16). A few months later, he inoculated the boy again, this time with matter from a fresh *smallpox* lesion. No disease developed. Jenner called this new procedure vaccination from the Latin word for cow *vacca* and cowpox *vaccinia* (16). The vaccine was met with great anticipation in England and Europe, where the annual death tolls were ~25,000 and 400,000, respectively (17). Despite its availability, deaths continued to occur from increased public concerns with safety and sanitation issues (18). Sharp falls in mortality in England did not occur until 1853 when parliament introduced the Vaccination Act, making smallpox vaccination in infants compulsory (16). By the 1860s, two-thirds of babies were vaccinated with great success, and parents were fined if their children were not vaccinated. The mandate, however, was met with violent protests in the streets (see quote above) and gave rise to antivaccination movements (12). These were fear campaigns built on vaccine-related deaths, new outbreaks and personal freedom violations, and were highly successful in raising doubt in people's minds (12–18).

As vaccine science and public messaging improved, smallpox and other deadly diseases, such as diphtheria, typhoid, and tuberculosis, began to decline in the 1920s (19). However, poliomyelitis was more resistant to vaccine design and it swept through towns of industrialized countries, paralyzing hundreds of thousands of children every year (19). In the 1950s, there were around 16,000 polio cases and ~1900 deaths each year in the USA. A polio vaccine was eventually developed in the early 1950s, and the mass inoculation of millions of US children dramatically reduced its prevalence by over 10-fold (19). Mistakes were made, new and improved vaccines were developed and, as a result of 80% global coverage each year (over 100 million infants), all three strains of polio have essentially been eliminated from the Western world (20, 21). However, small sporadic outbreaks still occur today in Afghanistan and Pakistan, which is being managed by the Global Polio Eradication Initiative (GPEI), whose goal is to achieve a polio-free world by 2025 (22).

Similarly, small outbreaks of mumps, rubella, and pertussis have occurred in under-vaccinated communities around the world (23, 24). Measles, another totally preventable disease, has flared up recently in the US, and other countries, from parents failing to vaccinate their children (25, 26). Before the measles vaccine, an estimated 3–4 million cases, 48,000 hospitalizations, and 450 deaths occurred annually in the US (25). The recent increase in vaccine hesitancy in the US has been largely driven by fear campaigns linking childhood vaccinations to autism (27, 28). There is no link. The negative campaigns continue to cite a 1998 study of Wakefield and colleagues, which was based on falsified data, and where the lead scientist received funding from lawyers acting for parents who were involved in lawsuits against vaccine manufacturers (29). The study was later retracted by the Lancet (29), and at least twelve follow-up studies, involving millions of children, have confirmed there is no association between vaccines and autism (28). These fear campaigns continue to threaten public health. In the US, in 1998 there were 89 cases of measles, and jumped over 14-times to 1,282 cases in 2019 (25, 26). Prevention is key and complacency is the killer.

Fast forward to the 2020 Covid-19 pandemic. From March 11 to Dec 20, 2020, there were 75 million reported cases globally, and 1.6 million deaths (3). The discovery and manufacture of multiple vaccines in a little over a year was nothing short of remarkable. The lead scientists Hamilton Bennett in the USA (Moderna), Ugur Sahin and Ozlem Tureci in Germany (Pfizer) and Sarah Gilbert in the UK (Astra Zeneca), devised new vaccines based on the science developed 20 years earlier for the related SARS and MERS viruses (30). The pharmaceutical companies then conducted randomized clinical trials with tens of thousands of patients and showed efficacy of 60–90% after the first dose, with very few adverse events (3). The new vaccines have saved many millions of lives and provide the most compelling reason for increasing investment in basic science (31).

Despite their success, vaccines are only beneficial if people get vaccinated. Early public polls indicated that ~70% would have the vaccine, ~20% expressed some doubt and ~10% were staunch objectors (32, 33). These are approximate percentages and depend upon country, demographics, race, ethnicity, religious beliefs, political ideology, and other factors [31*, 3*2]. However, the 20-percenters were genuinely concerned with safety and preferred a “wait-and-see” approach despite positive clinical trials (3). The 10 percenters, on the other hand, were more absolute and maintained multiple narratives including vaccines were unsafe, public messaging was false, the pharmaceutical industry was profit-driven, the science was fake, and the virus was no more deadly than the common cold (18, 34). Another argument is that vaccine mandates infringe upon personal rights and civil liberties (35). The majority of ethicists and lawyers argue that mandates are not infringements, particularly in a global health crisis (35). Personal liberties and rights don't allow you to drive down the wrong side of the road at 150 km/h. To date, only 60% of the total US population are fully vaccinated, and even after chilling appeals from anti-vaxxers, who have contracted the virus, it has made little difference to uptake (18, 36, 37). The Covid-19 pandemic has now become a “Pandemic of the Unvaccinated” contributing to over 97% of deaths in US hospitals (37).

The slow uptake to vaccinate in the USA and at least 70% of the world's population vastly increases the possibility of the emergence of more deadly variants, more unnecessary loss of life, increased mental health issues, exhaustion of healthcare systems, delays of goods and services, and destabilization of world's economies (38). The key point is the benefits of vaccination far outweigh the risks (3, 37). How do we shift thinking patterns of the undecided 20 percenters to take faster action? And how do we reach out to the small percentage of activists? We will return to this question later.

## Climate Change: Predictions Replaced by the Facts

Facts are stubborn things; and whatever may be our wishes, our inclinations, or the dictates of our passions, they cannot alter the state of facts and evidence.

John Adams *Argument of Defence* (39)

Climate change denial, like vaccine hesitancy, has a long history (4). The concept of global warming and greenhouse gases dates back 200 years to French mathematician Joseph Fourier (1768–1830). In 1820, Fourier reasoned that a fraction of the sun's heat energy was absorbed by the Earth's atmosphere, and acted like a garden greenhouse to keep the Earth's surface warm (Box 1). If there were no greenhouse gases, Fourier predicted the Earth would be frozen. In 1856, amateur scientist Eunice Foote (1819–1888), and a little later, physicist John Tyndall (1820–93) discovered that traces of gases and water vapor did indeed absorb heat, and suggested that CO_2_ may be responsible for the greenhouse effect (4). Tyndall wrote: “Thus the atmosphere admits of the entrance of the solar heat; but checks its exit, and the result is a tendency to accumulate heat at the surface of the planet” (40). *Foote's and Tyndall's experiments further predicted that tiny changes in CO*_2_ *levels could have a huge warming or cooling effect on Earth, and warmer air would hold more water vapor and add to the problem*. CO_2_ acted like sponge by absorbing multiple wavelengths of sunlight (40). These early views were met with high skepticism because how could such tiny changes in a gas, like CO_2_, influence the Earth's temperature on a global scale, a view that is still held by some climate denialists today.

In the late eighteenth century, Swedish chemist Svante Arrhenius entered the debate. He became intrigued with the century-old-problem of the Ice Ages (41, 42). After attending a lecture in 1894 by Swedish geologist Arvid Högbom (1857–1940), Arrhenius wondered if decreased volcanic activity might lower global CO_2_ levels and cool the Earth. *He focused on CO*_2_ *as a regulator, not water vapor, because the latter fluctuates daily (water cycle, clouds and rain), whereas CO*_2_ *was relatively fixed over geological timescales, mostly from volcanic activity* (7, 40, 41). Arrhenius calculated to a first approximation that if CO_2_ levels were halved, global temperatures would decrease by ~5°C, and possibly cause an Ice-Age; if they doubled, they would increase by the same amount, and warm the Earth (41). During his talk, Högbom also linked human activity to global warming, and calculated that human factories, and other industrial activities, were adding CO_2_ to the atmosphere at a rate that was comparable to natural processes (42). Importantly, Högbom's calculations did not prove that human-related CO_2_ emissions were warming Earth. It was a hypothesis that needed to be tested, and retested.

The scientific evidence for human activity warming the Earth began to accumulate in the 1930s and 1940s (42). English steam engineer Guy Stewart Callendar (1894–1964) published a landmark paper showing that doubling of CO_2_ could warm the Earth by 2°C, and that between 1900 and 1935, CO_2_ levels had risen by about 10%, implicating human activity (43). Callendar also showed a significant warming in the USA and North Atlantic regions after the industrial revolution, and after 20 years of measurements he concluded that a greenhouse-effect warming of the planet was already underway (43). Callendar wrote: “In the following paper I hope to show that such an influence is not only possible, but is actually occurring at the present time” (43). Most mainstream climate scientists, however, did not believe it and argued that human activities were too small to have a noticeable effect (42). With improved instruments and data collection, Callendar's work was later found to be surprisingly accurate, and it gained wide recognition (40, 42). This is science in action.

The next series of measurements linking fossil fuels to global warming came from US scientist Charles Keeling (1928–2005) (42, 44). Keeling was a chemist and tasked in the 1950s to painstakingly measure atmospheric CO_2_ and temperature at different places around the world, not only the US and Canada (44). In 1960, after compiling his data, Keeling generated his famous “Keeling curve” that showed a saw-tooth, steady, rise in CO_2_ levels over time (44). This dataset has been described by climate scientists as the single most important environmental advance in the twentieth century (40). Since that time, the “Keeling curve” has received overwhelming support from multiple lines of evidence, including CO_2_ measurements in ice cores obtained from the Greenland and Antarctic ice sheets (45).

Eventually, the world's political leaders began to listen to the climate scientists, and in 1997 organized the first meeting in Kyoto, Japan. The goal of the Kyoto protocol, as it became known, was to reduce greenhouse gases to 5.2% below the 1990 levels over the next decade. It failed because many of the world's largest and fastest growing economies, such as China, were not at the talks, and others did not ratify or withdrew (46). The next major effort was the 2015 Paris Climate Agreement in which 197 countries pledged to set their own targets to prevent a global temperature rise “well below” 2°C above pre-industrial times (47). It was soon realized a 2°C rise, however, was too high, and the critical temperature was lowered to 1.5°C, with the goal to:

- prevent small, low lying island states from sinking
- avoid the impacts of extreme weather
- limit the chances of an ice-free Arctic summer
- limit infectious diseases and their changing transmission trends.

In October 2018, the United Nations (UNs) Intergovernmental Panel on Climate Change endorsed the 1.5°C cap to “avert the most dire, irreversible consequences for the planet” (48). More recently, the urgency was ratchetted up by NASA's Gravity Recovery and Climate Experiment after they discovered that Greenland has lost ~279 billion tons of ice per year since 1993, and that Antarctica has lose ~148 billion tons per year over that same period (48). Glaciers continue to retreat almost everywhere around the world, including in the Alps, Himalayas, Andes, Rockies, Alaska, and Africa.

In an attempt to accelerate the Paris Agreement and the UN Framework Convention on Climate Change, the UK partnered with Italy and hosted the 26th UN Climate Change Conference of the Parties (COP26 UN) in Glasgow, November 2021 (47). The high-stakes conference brought together Governments responsible for 80% of global emissions, institutional investors, industry, the Global Citizen organization and social media outlets to instigate climate action, promote education and lower emissions (47). To push Governments harder, more than 700 institutional investors from across the globe signed an agreement to reduce emissions by 45% on 2010 levels by the end of 2030. *The conference agreed the situation is dire and predicted new patterns of infectious diseases and pandemics, affecting plants, animals, and humans and posing new risks for fresh water availability, food security and human health on a global scale* (47) (Box 2).

Box 2 **Code red—the facts.**

**Greenhouse gases, rising temperatures and the future**

- **CO**_**2**_ is typically 1,000 times more prevalent than other greenhouse gases.
- **CO**_**2**_ controls the world's thermostat, and is being turned up faster than at any time in the geological record (7).
- **1990**: the world emitted ~35 billion tons of greenhouse gases (in CO_2_ equivalents) into the atmosphere.
- **2000**: the 1990 value increased to ~37 billion tons per year (48).
- **2020**: the 2000 value jumped to 51 billion tons per year (a 35% increase in 20 years).
- **Emission contributors (%) from the things we do**:

~70% from fossil fuel energy use (electricity, heat, transport)
~22% from agriculture, forestry and land use
~5% from industry
~3% from waste.

- **2020:** China accounted for 26% of total global emissions, which was the same percentage as US, India, Russia, Japan combined (49).
- **2021**: July was the hottest on record since 1880, and devastating wildfires and floods occurred across the globe. Global surface temperature was 0.93°C (1.67°F) above the twentieth century average of 15.8°C (60.4°F) (47, 50).
- Glasgow's 26th UN Climate Change Conference of the Parties (COP26) pleaded with major industrialized countries that they must act now to reduce emissions so that 2050 targets can be reached (47).
- Delays and lack of commitment will lead to widespread and rapid changes in the atmosphere, ocean, cryosphere and biosphere, fires, floods, and infectious diseases (47, 49). These predictions are based on overwhelming scientific consensus (>97% of climate scientists).

Unfortunately, there were no breakthroughs (51). A week before the talks, Chinese President Xi Jinping called climate change a “wake-up call … to mankind”. This was an important acknowledgment because in 2016, greenhouse gas emissions from China alone accounted for 26% of total global emissions, about the same percentage as US, India, Russia, Japan combined (49). However, at the conference China (and India) rejected 2050 net-zero targets and offered no firm commitments to phase out coal-fired power to achieve carbon neutrality by 2050 (51). The USA, on the other hand, declared that it will lead the charge and promised to reduce emissions ~50% below 2005 levels by 2030, and become a net-zero emissions economy by no later than 2050. There is a lot of work ahead.

Having completed our brief history of vaccine hesitancy and global warming, it becomes clear that the denialists of both share much in common; they distrust science, they actively promote misinformation, they politicize and spread conspiracies, and they create their own “alternative” facts (Box 1) (6, 52, 53). It would be interesting to poll what percentage of climate denialists are vaccine denialists. It is my prediction given their shared narratives for rejecting scientific consensus, the percentage would be high.

### The Problem of Certainty and Science's Answer

Knowledge consists in the search for truth… It is not the search for certainty.

Karl R. Popper (1902–1994)

How do we decide on truth? This is a huge question that twentieth century philosopher Sir Karl Popper felt was the deepest reason for the fallibility of humans (11, 54). Popper argued that we cannot entertain finding absolute or certain truths, only repeated tests. Popper is correct, there is no method to obtain certain knowledge. I have argued elsewhere, this is not a “fallibility of humans”, but rather one of our greatest attributes because it drives new discovery and knowledge-building (11). The most powerful method to generate knowledge and problem-solve is science. Science begins with a question and ends with a question, with no absolutes (31). This open-ended nature of science creates much public confusion because it begs the question: How then does science explain the natural world and solves problems? This is at the heart of understanding science.

A scientist begins a study with a set of ideas or data from which an explanation or hypothesis is formulated. A grant is written and if funding is successful (5–10% success rate), the hypothesis can be tested using observation, experiment, measurement and statistical analysis. After completion, a structured manuscript is written and explanations and conclusions presented with references providing past ideas on the subject. The study, containing ethics approvals, funding sources and potential conflicts of interest, is sent off to a scientific journal where it is checked for suitability and peered reviewed. After review, if the conclusions are supported by the results, the editors may accept the study for publication. If independent reviewers find issues with the study, the editor may ask the authors to respond to the criticisms and resubmit a revised version. If one or more reviewers believe the study is flawed by design, or there are conflicts of interest, the editor will reject the paper. If accepted, the study's ‘truths' can be verified (or falsified) by others, and the new knowledge may change the way people think about new or old problems, predict new infectious diseases, identify environmental problems, or develop new therapeutics and technologies (31). Using this methodology, scientific discovery can lead to a better quality of life for all peoples living in a more sustainable world (31).

However, no matter how strong the scientific evidence, the facts are always under scrutiny. *This drives new thinking, new technologies, new knowing and new windows into human potentiality*. As mentioned, the scientific process of discovery does not deal with first causes, such as proving the existence of God. Science is not an enemy of faith—it simply cannot prove it one way or the other (11). Nor does science know with ‘absolute certainty' that all vaccines are safe, or that burning of fossil fuels causes global warming. *What matters is the preponderance of evidence*. The vast majority of actively publishing medical and climate scientists—perhaps as high 97%—agree that vaccines are safe (3, 55) and that human activity is increasing CO_2_ and warming the Earth's surface (50). *There are no absolute “truths” in science—only provisional truths generating provisional knowledge that is always subject to scrutiny*.

## Trust in Science and the Science Behind Doubt and Distrust

Different people in society may have different expectations of science, and therefore place different kinds of trust in science. … Trustworthiness can be earned, enhanced, or lost.

Resnik (56)

So far, we have discussed how science is based on evidence, however, for most people, it is based on trust (56, 57). Throughout history, science has excited our inner sensibilities with new advances in biology and medicine, discovery of a quasar, a black hole or a new butterfly species (11). However, high skepticism is triggered when the evidence challenges our personal belief systems, which continues today. This may include our personal views on the creation or evolution (11), or in the decision-making process of getting vaccinated or fighting climate change (58, 59). Although science was revered in the sixteenth and seventeenth centuries (9), it's power became firmly established with the invention of steam power and the industrial mechanization of production (11). Not everyone, however, welcomed “blue-sky” research. There is a wonderful story about English scientist Michael Faraday (1791–1867) who built the first electric motor in 1821 and electrical generator 10 years later. During a reported demonstration to the English Government in 1831, Faraday was asked by Sir William Gladstone (later to become Prime Minister) about the public usefulness of his research. “Why, sir,” replied Faraday, “one day you may tax it!” Today, this doubt sentiment remains active on controversial topics, with the science often propagated on social media as being untrustworthy or fake news (Box 1) (8, 10, 58–60).

**Figure 1:**
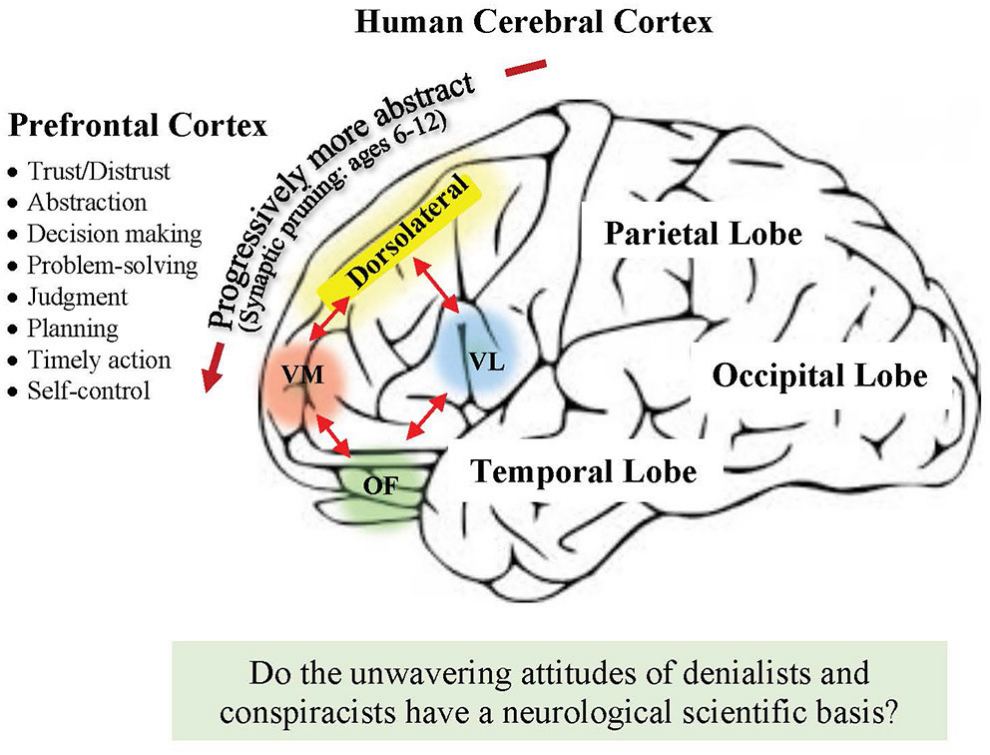
Schematic of the human brain and prefrontal cortex (PFC) which constitutes more than 25% of the entire cerebral cortex. The PFC is where executive functions are carried out and depend on working memory, flexible thinking, and self-control (61). Accepting, doubting or distrusting ideas are believed to occur largely in the dorsolateral region, which communicates with the ventromedial (VM), ventrolateral (VL) and orbitofrontal (OF), and other parts of the brain, which all play a role in controlling our personal beliefs and behavior (61, 62). The development of abstract thinking is believed to occur during childhood from the age of *six* and involves the anteriorization of circuitry and synaptic pruning (63–65). This early anteriorization may provide a new window of opportunity for teaching how to distinguish truth from falsehood in our educational systems (see text).

Distrusting new facts has a scientific basis. It is believed to involve activation of the brain's dorsolateral prefrontal cortex (DPFC) region, which is responsible for suppressing unwanted representations (Figure 1) (5, 66–68). When confronted with new ideas that strongly contradict one's beliefs, the dorsolateral prefrontal cortex is activated, and initially leads to criticism or suppression of those new ideas (67, 68). This is a normal human reaction because trust and distrust are essential components of social success, and from an evolutionary perspective must have been under high selection pressure to bring about change (61, 67). With respect to trust in science, a recent study involving 120,000 respondents across 126 countries showed that people are more likely to get vaccinated where trust in science is high (57), and probably the same is true for global warming.

At the molecular level, how human thinking patterns in the prefrontal cortex switch from doubt to belief, and belief to action remain a deep mystery. Once believed to be cognitively “silent,” the region has many interconnections with other parts of the brain including the amygdala, hypothalamus, midbrain, and pons, which all play a role in controlling our personal beliefs, feeling of contributing, decision making and behavior (Figure 1) (5, 62, 66). People's differences in control of trust, distrust and false beliefs appear to be localized to circuits in different prefrontal cortical regions (Figure 1) (68, 69). False beliefs are common, for example, in neurodegenerative disorders, particularly dementia with Lewy bodies and frontotemporal dementia, and in a number of psychiatric disorders (68). Asp and colleagues studied ten patients with bilateral damage to the ventromedial prefrontal cortex (VMPFC) and found they had significantly higher resistance to authoritarian persuasion and religious fundamentalism compared to 10 patients that had suffered neurological damage outside the region (70). Interestingly, in those patients with VMPFC damage, specific religious beliefs increased in intensity after brain injury. The research group further presented evidence that prefrontal-mediated doubting may explain some biases of intuitive judgments (71). Admittedly, these studies have small sample sizes with low statistical power, however, they encourage further scientific investigation. On the other side of the spectrum, studies on delusional beliefs *in healthy subjects* appear to show they have neuropsychological origins, which reside in the right DPFC (72, 73). The possibility exists, therefore, that the unwavering attitudes of anti-vaxxers and climate denialists and conspiracists may have a neurological basis (Figure 1). The unwavering attitudes may reside in specific functional circuits of the prefrontal cortex, which are more resistant to change and perpetuated by social media. Since the brain is highly plastic (Box 1), one could hypothesize that reinforcement of such fixed false ideas could build stronger local circuitry, with stronger unwavering opinions. If true, it means that the reverse may be possible with proper education and public messaging.

## Possible Solutions to Escalating Problems: A Call to Action

Social media has also been weaponized against the public health community to spread disinformation and misinformation, and the public health community has yet to devise a successful strategy to mitigate this destructive use of social media.

Moore et al. (74)

Two possible solutions to address misinformation on vaccine hesitancy and climate change are:

- Education and improved public messaging
- Regulation of social media content.

### Education and Improved Messaging

New education programs are urgently required to understand how to distinguish truth from falsehood that begin at preschool and continue through to primary and high school, and university (75). In addition, improved public campaigns for all ages are required to promote scientific literacy (8). A better understanding of how science works is key, and the problem of certainty must be addressed. Fit-for-purpose programs in schools could include five real-time conspiracies: 1) Vaccines-anti-vaxxers, 2) global warming-climate denialism, 3) the flat Earth, 4) the moon landing was a hoax, and 5) the Earth is 6,000 years old. Understanding and debunking falsehoods could be individual and group exercises with an emphasis on distinguishing fact vs. opinion, having some kind of test and evidence, and how a consensus could be reached in deciding truth (75, 76). New programs should begin early to align with children transitioning their thinking skills from concrete representational to more abstract learning, a process that begins around 6 years old and involves synaptic pruning and anteriorization of circuitry in the prefrontal cortex (Figure 1) (63–65). The possibility exists that during this pruning phase, it may highly beneficial and transformative in a child's education to develop a new skill-set on the importance of “lazy” thinking vs. “accurate” thinking when confronted with new information. Admittedly, this proposal needs testing and would take a decade or so for the social benefits to be realized.

### Regulation of Social Media

Given that social media platforms have been allowed to self-regulate for decades, some regulation is urgently required to prevent the constant barrage of misinformation entering our lives (77, 78). Regulation will not be easy to implement because the Big Three, Google, Facebook and Twitter connect ~50% of the world's population (~4 billion users), and they have strong liability protections. In contrast, traditional media outlets, such as newspaper, radio, television or cable, are regulated with editorial oversight to control content. However, in comparison they have a minuscule footprint. The New York times, for example, in 2019 had ~8 million print and digital subscribers. In the same year on social media: 188 million emails were exchanged, 511,000 Tweets were posted, 4.5 billion videos were being watched, 390,030 Apps were downloaded, 510,000 comments were posted on Facebook, and a staggering 3.5 billion Google searches were made every minute (79). It is worth repeating. Each activity occurred every 1 minute. This equates to an estimated 2.5 quintillion (1 followed by 18 zeros) bytes of personal data that are created on social media every day (79). These statistics are mind-boggling and unprecedented in human history.

Despite early good intentions to connect people, these massive social media platforms have become a danger to society (80). Their current algorithms have empowered advertisers, scammers, conspirators, influencers, foreign adversaries and trolls to target individuals and groups, and use their personal data to feed into perpetuating false or misleading information on any topic (77, 78). Without the appropriate checks and balances, a society cannot function with the constant barrage of misinformation. Facts matter.

In 2021, a whistleblower told US Congress that Facebook's own research showed that certain algorithms were harming the mental health and body image of children and teens, including promoting dangerous behaviors, such as eating disorders (81). Nothing was done. More clicks equal more advertising, and more advertising means higher profits (82, 83). Social media users provide an enormous amount of “unprotected” personal data, which feed into algorithms that link the user to potentially millions of like-minded individuals and perpetuate dangerous falsehoods and fake news (Box 1) (83, 84).

Ironically, it has been the “tiny” traditional media outlets who have served as the public watchdogs over social media by calling out their false or harmful content (81). However, the nominal changes made by social media appear to be pre-emptive to avoid Government oversight. Perhaps the formation of a World Social Media Organization (WSMO), made up of government and industry partners from around the world, is required to bring about real change. Such a body could be analogous to the WHO for global health. The important point is disagreement is to be encouraged; it is when false or misleading information is knowingly used to manipulate individual or public opinion, it becomes a problem. If we continue to enable the current practices of perpetuating lies and deception, without fact checking, historians 100 years from now may write: “people of the early twenty-first century became so overwhelmed with digital information that they failed to develop the skills to sufficiently process it to the detriment of their health and society.”

## Conclusions

Understanding how science works is key for making informed decisions about vaccination safety and climate change urgency. In recent years, there has been a growing distrust in science perpetuated by social media. Distrust appears to be amplified when the evidence calls for personal decision-making and action. In a rapidly changing world, it is important to embrace robust debate, listen to the experts in their respective fields, verify what is said, and not be swayed by the denialists or conspirators. What matters is the preponderance of scientific evidence. The unwavering attitudes of anti-vaxxers and climate denialists may have a neurological basis, and the specific neural circuits may be consolidated or strengthened by social media misinformation. Education and improved public messaging are key, and social media providers require regulatory oversight to remove the algorithms that deliberately spread harmful/dangerous content.

## Author Contributions

The author confirms being the sole contributor of this work and has approved it for publication.

## Conflict of Interest

The author declares that the research was conducted in the absence of any commercial or financial relationships that could be construed as a potential conflict of interest.

## Publisher's Note

All claims expressed in this article are solely those of the authors and do not necessarily represent those of their affiliated organizations, or those of the publisher, the editors and the reviewers. Any product that may be evaluated in this article, or claim that may be made by its manufacturer, is not guaranteed or endorsed by the publisher.
